# Contrast Effect of Facial Attractiveness in Groups

**DOI:** 10.3389/fpsyg.2020.02258

**Published:** 2020-09-15

**Authors:** Yatian Lei, Xianyou He, Tingting Zhao, Zuye Tian

**Affiliations:** ^1^ School of Psychology, South China Normal University, Guangzhou, China; ^2^ Center for Studies of Psychological Application, South China Normal University, Guangzhou, China; ^3^ Guangdong Key Laboratory of Mental Health and Cognitive Science, South China Normal University, Guangzhou, China; ^4^ Key Laboratory of Brain, Cognition and Education Sciences (South China Normal University), Ministry of Education, Guangzhou, China; ^5^ School of Health Management, Guangzhou Medical University, Guangzhou, China

**Keywords:** facial attractiveness, group, special face, the cheerleader effect, contrast effect

## Abstract

Research on facial attractiveness is an important part of aesthetics. Most relevant studies in the area have focused on the influence of individual perspectives on facial attractiveness, but it is necessary to consider the effect of contextual information on facial attractiveness. In this study, we examine the influence on attractiveness of special faces in a given group. We define a “special face” as one that is significantly different from other members of the same group in terms of facial attractiveness. We conducted three experiments to explore the influence of different modes of presentation and central positions in a group on the judgment of attractiveness of the special face. The results show the following: (1) When the special face was part of a given group, the subjects made more extreme judgments than without it: that is, they judged the most attractive face as more attractive and the least as less attractive than when faces were presented alone. (2) The subjects rated the most attractive faces lower and the least attractive faces higher when the target faces in the middle of the group than in other positions. The results favored the contrast effect: when the subjects judged the attractiveness of target stimulus, they always compared it with the environment, which then became a reference in this regard. Moreover, the greater the amount of contextual information perceived, the higher the likelihood that assimilation would occur.

## Introduction

Facial attractiveness has traditionally been a key area of research on aesthetics. Research has led to a better understanding of the variables that influence human facial attractiveness. However, it is not sufficient for researchers to study only features of the attractiveness of an individual because society can be considered as being composed of groups. We live with our families, study with our classmates, and work with our colleagues. Most of the time, we are with groups. Therefore, it is necessary to study the influence of groups on facial attractiveness. We thus need to expand this examination to the group level to explore factors influencing facial attractiveness in this domain.

Studies have shown that the evaluation of a target can either be assimilative or contrastive given its context (Mussweiler, 2001). A perceiver’s focus on similarities or differences between a target and its context leads to assimilative or contrastive judgments (Mussweiler, 2001; Ruys et al., 2006). When the characteristics of the target stimulus are perceived to be similar to that of the surrounding context, assimilative judgments may be formulated – for example, that a face considered attractive on average is considered more attractive within a group of attractive faces (Geiselman et al., 1984). On the contrary, when a target stimulus is perceived as different from the context, contrastive judgments may occur – for example, an unattractive face is considered to be less attractive within a group of attractive faces (Melamed and Moss, 1975).

The emergence of contrast and assimilation effects is influenced by such variables as the quantity, layout, and order of presentation of objects (Abele and Petzold, 1998), and whether the context and the target are considered to be part of the same category or group (Meyers-Levy and Sternthal, 1993). In early studies in the area, researchers had the participant’s view and rate portraits in the context of attractive and unattractive impressions. Contrast was observed when pairs of persons were considered unrelated. However, assimilation occurred when they were considered as friends. Assimilation effects were observed when two faces were presented simultaneously (simultaneous assimilation), and contrast effects were noted when the faces were presented in sequences (successive contrast; Wedell et al., 1987).

While assimilation effects have been reported when faces are presented simultaneously (Geiselman et al., 1984; Wedell et al., 1987), contrast effects have also been consistently observed (Tousignant, 2016). Moreover, Rodway et al. (2013) found that the central position of an attractive face, in a group of faces, also influenced the degree of assimilation and contrast with the surrounding faces.

Furl used normalization models to predict how the evaluation of facial attractiveness depends on context (Furl, 2016). Two attractive or average targets were simultaneously presented along with an undesirable distractor, the attractiveness or averageness of which was changed in each trial to manipulate the range of attractiveness or averageness of the faces. The participants were instructed to select the most attractive or average face among three faces. The contrast effects occurred when the participants observed faces with different degrees of attractiveness. Normalization represented the neural activity of the brain, and can be regarded as a putative typical neural computation (Carandini and Heeger, 2012).

There is a phenomenon, whereby an individual is considered more attractive when in a group than individually, called the *cheerleader effect* (Rashid and Fryman, 2008) or the *friend effect* (Ying et al., 2019). Walker and Vul (2014) selected 100 photos of single males and females, each of whom featured three people. Their faces were presented in a group photo, and individual photos clipped from them. The subjects were required to judge the attractiveness of all faces. The results showed that the facial attractiveness ratings of an individual in a group photo were higher than that in an isolated portrait. The researchers then shortened the duration of presentation, increased group size, and blurred the photos. The ensuing results were nearly identical to those before, with a smaller difference. The researchers suggested that the cheerleader effect arises from interactions between the ensemble coding in the visual system and the ensemble averageness of group members. When we observe an array of stimuli at the same time, our brains involuntarily perceive statistics of the ensemble of the information (Haberman and Whitney, 2007; Alvarez, 2011; Whitney and Leib, 2018).

Ensemble coding enables people to cursorily extract the ensemble statistics of objects (Haberman and Whitney, 2007). People can perceive the summary information of a large crowd regarding motion (Watamaniuk et al., 1989), average size (Ariely, 2001; Chong and Treisman, 2005), average orientation (Parkes et al., 2001; Ross and Burr, 2008), and even the average emotional expression of faces (Haberman and Whitney, 2009) as well as moving bodies (Sweeny et al., 2013). Because of the high accuracy of ensemble coding, observers have been able to extracted the characteristics of a group better than those of an individual (Sweeny et al., 2013), such as the emotion reflected in faces (Elias et al., 2017).

Ensemble statistics also allow people to obtain a gist of the attractiveness of a group of faces presented simultaneously or sequentially (Haberman and Whitney, 2007, 2009; Sweeny and Whitney, 2014; Ying and Xu, 2017; Whitney and Leib, 2018). The brain seems to be able to perceive the average facial attractiveness of a group involuntarily, thus influencing the judgment of the target faces implicitly (Ying et al., 2019). Ying and his colleagues have shown that “two distinct levels of ensemble statistics can occur for the same facial trait: the gist averaging during static spatial ensemble coding, and the morph averaging for temporal ensemble coding” (Ying et al., 2020, p. 12).

In case of a highly attractive face in a group, people’s facial attractiveness scores for the group are higher than the average score of the attractiveness of group members, which are referred to as the group attractiveness effect (van Osch et al., 2015). The principle of this effect is that when the duration of presentation is short, the subjects selectively focus on the most attractive faces in the group, which produces a memory effect: the individuals most concerned are remembered better. Thus, the most attractive member has an impact on the attractiveness of the entire group. It has been shown that the visual processing mechanism of people’s observations of a group is not always ensemble coding. In special circumstances, people may pay attention to the special individual in the group, followed by the entire group, which is more akin to selective attention (van Osch et al., 2015).

There are two different views on the contradiction between ensemble coding and selective attention. Gestalt psychologists argue that ensemble coding is a specific form of Gestalt processing. Because the processing of group faces is an ensemble, this should be assigned an overall priority, which means that before more special features are processed than otherwise (Navon, 1977). However, researchers who support selective attention believe that ensemble coding does not hold true for all perceptual processes. Although these holistic features occupy the primary place in visual processing, ensemble coding cannot explain the principle of processing of the effect of group attractiveness. In addition, Gestalt psychologists focus only on abstract and simple nonsocial stimuli, and cannot provide a reasonable explanation for complex social stimuli, such as social groups and their processing mechanisms. Thus, ensemble coding is not sufficient to explain the principle of processing of the facial attractiveness of varieties of groups (van Osch et al., 2015).

Researchers have claimed that the effect of group attractiveness is similar to the bias in sample size in decision making; the greater the number of stimuli in a group is the better is the judgment of the average. People’s judgments on the average values of targets are more likely to biased upward when these targets in a larger group (Price et al., 2006, 2014), which is an extreme method of judgment. These findings are similar to those by van Osch et al. (2015) who showed that the larger is the group of individuals, the larger is the group attractiveness effect. Maner et al. (2003) found that when 15 faces were simultaneously presented to subjects, the number of attractive faces in the group was overestimated. Another study increased the cognitive load on subjects in experiments on rating the facial attractiveness of a group of people, and the results showed that the subjects overestimated the number of faces with extreme characteristics (Rothbart et al., 1978). This means that the group did not use only ensemble coding for visual processing. In case of faces with special characteristics in a given group, people attended more to them, and this might also have had an impact on the results for the entire group.

A person’s facial attractiveness in a social context may be influenced by his/her position in a group (Rodway et al., 2013). According to the cultural norms of Western society, people in the middle of a group are generally the most important in the group. Thus, in most social situations, people in the most esteemed social position are in the middle of the group. In another experiment, people were shown photos of five candidates who had applied for an internship and asked to select one (Raghubir and Valenzuela, 2006). The results showed that the participants were more likely to choose the person whose photo was shown to them in the middle than at the beginning or the end. These two experiments also show that people are more likely to pay attention to individuals in the middle in a group.

Some researchers have found that an individual positioned in the middle of a given group has a higher relative position and social standing (Taylor and Fiske, 1975; Raghubir and Valenzuela, 2006; Valenzuela and Raghubir, 2009). This is because of the specificity of this position; an observer has implicit expectations of the importance of objects in the middle (McArthur and Post, 1977; Raghubir and Valenzuela, 2006; Valenzuela and Raghubir, 2009). Previous research has shown that knowledge of someone’s higher social status can improve the observers’ evaluation of the attractiveness of their faces (Kalick, 1988; Kowner, 1996; Chu et al., 2007; Dunn and Searle, 2010).

Walker and Vul (2014) claimed that the cheerleader effect contributes to explaining the processing of the attractiveness of faces in a group, but the experimental design that they used to establish this conclusion has shortcomings. Their research did not consider the difference between the target face and other faces. When people compared the target face with other faces, their evaluation was influenced by the latter. Individuals also attend to differences or similarities between the stimulus object and the surrounding stimuli. The assimilation effect is generated when the characteristics are similar, and the contrast effect is generated when they are different (Bless and Schwarz, 2010).

When studying the facial attractiveness of a group, the location of the target faces is an important factor. Some research on the effect of position has yielded different findings from the above (Rodway et al., 2013; Carragher et al., 2018). Rodway et al. (2013) found that the attractiveness of the middle face in a group depends on the contrast effect or the assimilation effect, but Carragher et al. (2018) found that this position did not influence the cheerleader effect. There were important differences in experimental design between the studies, such as in terms of the procedure, number, and size of faces displayed, and between the facial attractiveness of the target and the context.

This study manipulates the facial attractiveness of the target (high/low, defined as special faces) and its position (middle/random) to explore whether the cheerleading effect is still observed when its attractiveness is rated in different situations.

We propose the following hypotheses: (1) When the target faces are presented in a group, the subjects make more extreme judgments than they do when the faces are presented to them separately. That is, the score assigned to the most attractive face is higher and that assigned to the least attractive face is lower than otherwise. (2) The middle influences the evaluation of the target face in a group.

## Experiment 1

### Methods

#### Participants and Design

The purpose was to explore whether an individual’s judgment of the attractiveness of a particular face is influenced by its mode of presentation. A 2 (facial attractiveness: high vs. low) × 2 (mode of presentation: in an isolated portrait vs. in a group) intra-subject design was used. The attractiveness score was the dependent variable. A total of 30 participants were recruited (20 males). They all had an undergraduate degree or a higher educational qualification, ranged from 18 to 35 years of age, were right-handed, and had normal vision.

#### Materials

Five hundred facial pictures were selected from the Internet; half of males and the other half of females, by querying the Baidu search engine (Li et al., 2019). The search term was “Chinese face images.” We selected images where the subject directly faced the camera, and had both eyes directed toward it. All subjects appeared to be of Asian ethnicity, and exhibited neutral expressions. Photoshop was used to process all pictures, and only the facial features were preserved while controlling for confounding elements (e.g., skin texture and hair color). All images were resized to 260 × 280 pixels and presented on a black background. The factors that might have affected the participants’ responses, such as the grayscale and color, also were balanced.

In a pilot study, 60 college students (22 males) were recruited to assess the attractiveness, emotions, and familiarity of all facial images on a seven-point scoring method. For the dimension of attractiveness, 1 was “very low” and 7 was “very high”; on the dimension of emotion, 1 was “very negative” and 7 was “very positive”; for familiarity, 1 was “very strange” and 7 was “very familiar.” A total of 320 faces with low familiarity (*M* = 2.69, *SD* = 0.29) and neutral emotions (*M* = 3.64, *SD* = 0.63) were selected as formal experimental materials, and were sorted according to attractiveness score. The 25 most attractive (*M* = 4.63, *SD* = 0.2) and the 25 most unattractive faces (*M* = 2.41, *SD* = 0.17) were selected for each gender (in the section Supplementary Material). There were significant differences in facial attractiveness between the groups, *t*(59) = 16.02, *p* < 0.001, and none in terms of familiarity, *t*(59) = 1.53, *p* > 0.05, or emotion, *t*(59) = 1.87, *p* > 0.05. The other 220 faces with average attractiveness were used as the surrounding faces (*M* = 3.42, *SD* = 0.54). Because the attractiveness of other faces when presented in a group differed significantly from those of the most and least attractive faces, the selected faces were defined as “special faces” to emphasize the difference in their attractiveness of the other faces. The “special faces” were used as target faces, and the other faces were used as surrounding faces.

#### Procedures

**Figure 1:**
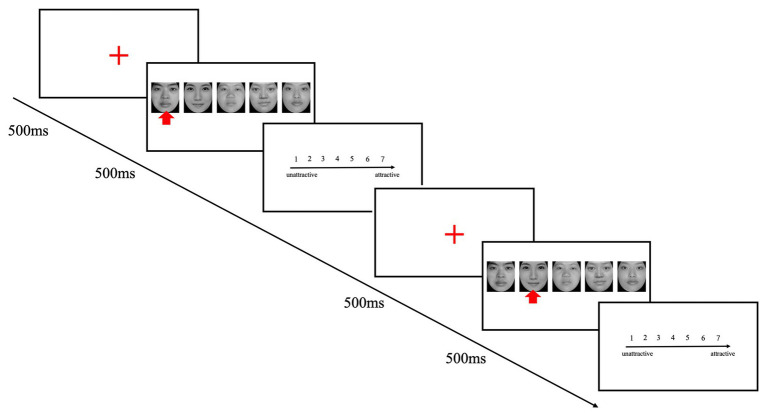
A seven-point scale and stimuli used in all experiments. The participants rated the attractiveness of the target faces twice: once in a group photograph (the arrow indicates the face was to be rated) and once separately. The faces images were selected from an online public website. + represents a red fixation cross.

As shown in Figure 1, this experiment consisted of two stages: face presentation and face evaluation. Once the participants had been instructed on the procedure, a 500 ms display of a red fixation cross was presented at the center of the screen. Once the fixation point had disappeared, a facial image was presented on the screen for 500 ms, and the participants were then asked to assess its attractiveness. The score was in the range 1–7, where 1 designated the least attractive face, 7 the most attractive one, and there was no limit to the reaction time. Following the assessment, the subject pressed a key to move on to the next trial.

The experiment featured two blocks: a single-face block and a block of faces in a group. In the single-face block, only one face was presented as a baseline block; in the block of faces in a group, five faces of people of the same sex were presented, where one of them, placed randomly, was the target face. To avoid the order and repetition effects, the two blocks in the experiment were balanced: 50 of the most attractive (25 female) and least attractive (25 female) faces were rated twice: once in an isolated portrait and again in a group photo. There were 200 trials in total, with 100 in each of the two blocks. The experiment lasted for 10–15 min.

#### Results and Discussion

**Figure 2:**
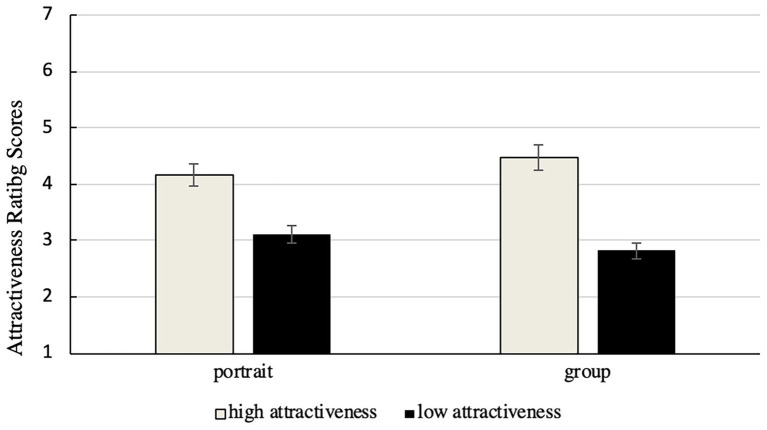
The scores of special faces (attractive and unattractive faces) in different modes of presentation. The error bars represent standard intra-subject error.

SPSS17.0 software was used for the two-factor analysis of variance (ANOVA). The average results of the ratings assigned by all participants to the target faces in different modes of presentation are shown in Figure 2.

The ANOVA showed that the main effect of the modes of presentation was not significant, *F*(1, 29) = 0.17, *p* = 0.68, that of facial attractiveness was significant, *F*(1, 29) = 110.87, *p* < 0.001, *η* ^2^ = 0.79, and the scores assigned to attractive faces were higher than those assigned to unattractive faces. The interaction effect was significant, *F*(1, 29) = 10.55, *p* < 0.001, *η* ^2^ = 0.27. The attractiveness scores assigned to attractive faces in group presentation (*M* = 4.48, *SD* = 0.43) were significantly higher than those assigned to each of them in an isolated portrait (*M* = 4.16, *SD* = 0.54), *F*(1, 29) = 9.09, *p* < 0.01, *η* ^2^ = 0.24, whereas those of unattractive target faces in group presentation (*M* = 2.82, *SD* = 0.61) were significantly lower than in isolated portraits (*M* = 3.11, *SD* = 0.61), *F*(1, 29) = 4.74, *p* < 0.05, *η* ^2^ = 0.14.

The results show that when special (most attractive and unattractive) faces appeared in a group, the observers made more extreme evaluations, where this show that the attractiveness scores of the attractive special faces were higher, and those of the unattractive special faces were lower.

The cheerleader effect can be quantified as the difference between the scores assigned in the experimental condition (in a group photo) and the baseline (in an isolated photo). In the results, the most attractive faces were rated to be more attractive, while the most unattractive faces were rated as less attractive than the baseline. The results for the attractive target faces replicated the cheerleader effect, whereby the scores of the target face in group presentation should have been higher than that in an isolated picture. However, the results for unattractive faces were inconsistent with the cheerleader effect. The results provided evidence supporting the contrast effect.

**Figure 3:**
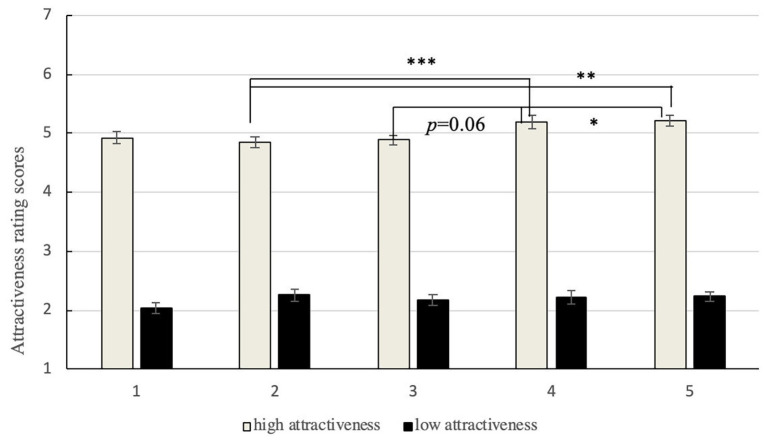
Mean attractiveness ratings at each position for attractive and unattractive target faces. The error bars represent standard intra-subject error.

We wondered if the results had been obtained because of the positions of the faces in groups. Researchers have found that observers look at objects in the middle first, for longer, and more frequently (Tatler, 2007; Bindemann, 2010). It is plausible to assume that special attractive faces in the middle would have been rated more highly. A 2 × 5 [attractiveness (high vs. low) × position (1, 2, 3, 4, and 5)] repeated-measure ANOVA was conducted on the mean ratings of facial attractiveness to examine if the positions of the faces affected the attractiveness ratings (in Figure 3).

This revealed a significant effect of position, *F*(4, 116) = 3.862, *p* = 0.006, *η* ^2^ = 0.12, and the interaction between attractiveness and position reached significance, *F*(4, 116) = 3.48, *p* = 0.01, *η* ^2^ = 0.11.

The analysis of the main effect showed that position influenced the ratings of highly attractive faces (*p* < 0.001), but not the unattractive ones (*p* > 0.05). Further analysis showed that the attractive target faces obtained the highest scores when presented in position 4 (*M* = 5.19, *SD* = 0.58) or 5 (*M* = 5.22, *SD* = 0.48) and the lowest when presented in position 2 (*M* = 4.84, *SD* = 0.52) or 3 (*M* = 4.89, *SD* = 0.41).

On the contrary, the ratings of the most attractive faces decreased significantly when presented in the middle of other, less attractive, faces, indicating support for assimilation. This is in line with predictions that position affects the perceived attractiveness of an attractive target face. The results indicate that the evaluation of facial attractiveness may rely on the relationship between the target face and surrounding faces, and that except for the positive effects found in past research, the middle can have a negative influence on attractiveness.

No significant difference was observed in the attractiveness ratings of the unattractive target faces in different positions. This might have been the case because people do not prefer unattractive faces, because of which the participants did not attend to them in any position. This possibility was explored in subsequent experiments.

## Experiment 2

Experiment 1 showed the contrast effect in groups and the effect of position on the perceived attractiveness of faces. In this experiment, the position of presentation was used as an inter-subject factor to test whether a target face in the middle is perceived as more attractive when participants are asked to focus on this position.

### Methods

#### Participants and Design

This experiment was designed to explore whether an individual’s judgments of the attractiveness of a particular face is influenced by its position of presentation. A 2 (attractiveness: high vs. low) × 2 (presentation position: middle vs. random) mixed design was used. Facial attractiveness was the intra-subject variable, and the position of presentation was the inter-subject variable. The attractiveness score was the dependent variable. Fifty-eight participants were recruited (34 males), who had not participated in Experiment 1. All had an undergraduate degree or higher, were from 18 to 35 years old, were right-handed, and had normal vision.

#### Procedure

We used the same lab settings, procedure, and faces as in Experiment 1 with a few changes. All faces were presented in groups. The subjects were randomly divided into a “Middle” group and a “Random” group. Participants in “Middle” group judged only the faces presented in the middle, and those in the “Random” group judged the faces presented in random positions except for the middle in a group photo.

#### Results and Discussion

**Figure 4:**
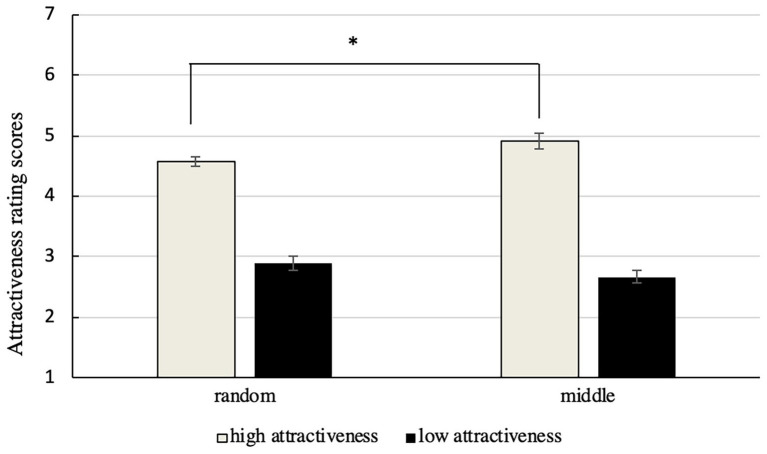
Scores assigned to highly attractive and unattractive faces in different positions of presentation. The error bars represent standard error.

SPSS17.0 was used to analyze the two-factor repeated-measure ANOVA. The scores of special faces (highly attractive and unattractive faces) in different positions of presentation are shown in Figure 4.

The results show that the main effect of facial attractiveness was significant, *F*(1, 56) = 365.77, *p* < 0.001, *η* ^2^ = 0.97, which means that scores assigned to highly attractive faces were significantly higher than those assigned to highly unattractive faces. The main effect of position was not significant, *F*(1, 56) = 0.23, *p* = 0.63, whereas the interaction effect was, *F*(1, 56) = 8.15, *p* < 0.01, *η* ^2^ = 0.13.

The results of simple effect analysis show that scores assigned to highly attractive faces when presented in the middle (*M* = 4.92, *SD* = 0.7) were significantly higher than those assigned when they were in a random position (*M* = 4.57, *SD* = 0.41), *F*(1, 56) = 5.36, *p* < 0.05, *η* ^2^ = 0.09, whereas no significant difference was observed between the scores assigned to highly unattractive faces when presented in the middle (*M* = 2.67, *SD* = 0.62) and in a random position (*M* = 2.9, *SD* = 0.69), *F*(1, 56) = 1.85, *p* = 0.18.

The results indicate that when the special faces were presented in the middle, the subjects’ scores of attractive faces were higher than when they were in a random position, but the differences in scores assigned to unattractive faces were not significant.

The results partially replicated the findings of Experiment 1. Once again, the perceived attractiveness of unattractive targets was not significantly influenced by their position in the group. Inconsistently with the Experiment 1, the observers considered attractive faces in the middle to be even more attractive. This might have occurred because people tend to focus on the middle in any scene. The longer one looks at a target face, the more attractive it is perceived to be. This is called the exposure effect (Moreland and Beach, 1992). Whether the time spent looking at a target face affects its perceived attractiveness was examined in the next experiment.

## Experiment 3

In this experiment, we increased the duration of the subject’s gaze on the target face. We used the same lab settings, procedure, and faces as in Experiment 1, but the duration for which images were shown was doubled to 1,000 ms.

### Methods

#### Participants and Design

A 2 (attractiveness: high vs. low) × 6 [presentation position: in a group (1, 2, 3, 4, and 5) vs. alone] intra-subject design was used. The attractiveness score was the dependent variable. Thirty participants were recruited (12 males), who had not participated in Experiment 1 or 2. All had an undergraduate degree or higher, were from 18 to 35 years of age, were right-handed, and had normal vision.

#### Results and Discussion

**Figure 5:**
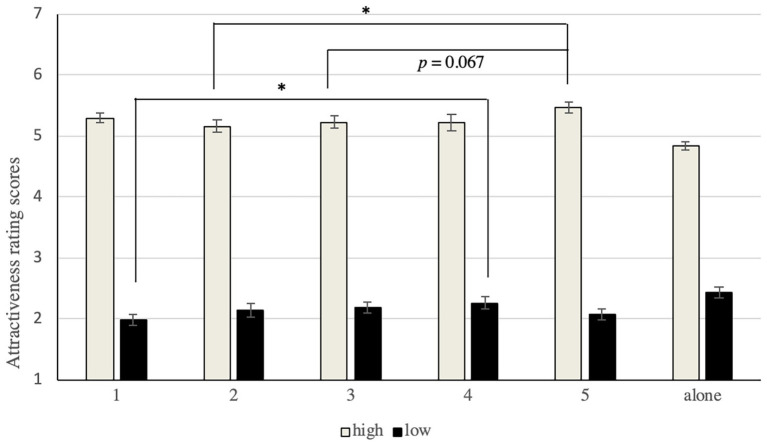
Scores of highly attractive and unattractive faces in different positions of presentation. The error bars represent standard intra-subject error.

SPSS17.0 was used to analyze the two-factor repeated-measure ANOVA. Two participants quit during the experiment and their data were removed. The scores of special faces (highly attractive and unattractive faces) in different positions of presentation are shown in Figure 5.

A 2 × 2 [attractiveness (high vs. low) × position (in a group vs. alone)] repeated-measure ANOVA was conducted on the mean ratings of attractiveness of the faces. The results show that the main effect of facial attractiveness was significant, *F*(1, 27) = 1511.13, *p* < 0.001, *η* ^2^ = 0.98, which meant that scores assigned to the highly attractive faces were significantly higher than those given to unattractive faces. The main effect of position was not significant, *F*(1, 27) = 2.34, *p* = 0.14, but the interaction effect was, *F*(1, 27) = 89.33, *p* < 0.01, *η* ^2^ = 0.77.

The results of the main effect analysis show that the scores of highly attractive faces presented in a group (*M* = 5.28, *SD* = 0.43) were significantly higher than when presented alone (*M* = 4.84, *SD* = 0.34), *F*(1, 27) = 53.55, *p* < 0.001, and those of unattractive faces presented in a group (*M* = 2.14, *SD* = 0.41) were significantly lower than when presented alone (*M* = 2.44, *SD* = 0.51), *F*(1, 27) = 23.38, *p* < 0.001.

A 2 × 5 [attractiveness (high vs. low) × position in a group (1, 2, 3, 4, and 5)] repeated-measure ANOVA was conducted on the mean ratings of the attractiveness of faces. The results show that the main effect of facial attractiveness was significant, *F*(1, 27) = 1260.17, *p* < 0.001, *η* ^2^ = 0.98, which meant that scores assigned to highly attractive faces were significantly higher than those given to unattractive faces. The main effect of position was not significant, *F*(4, 108) = 1.50, *p* = 0.21, but that of the interaction effect was, *F*(4, 108) = 5.23, *p* < 0.01, *η* ^2^ = 0.16.

The results of the main effect analysis showed that the position influenced the ratings of highly attractive [*F*(4, 108) = 3.09, *p* < 0.05] and unattractive faces [*F*(4, 108) = 3.39, *p* < 0.05]. A *post-hoc* comparisons showed that the ratings of attractive target faces presented at position 5 (*M* = 5.469, *SD* = 0.421) were significantly higher than those presented at position 2 (*M* = 5.16, *SD* = 0.54), *t* = 3.67, *p* < 0.05, and marginally higher than position 3 (*M* = 5.23, *SD* = 0.55), *t* = 3.67, *p* = 0.066. A *post-hoc* comparison of the unattractive faces showed that the ratings of the target faces presented at position 4 (*M* = 2.26, *SD* = 0.54) were significantly higher than those presented at position 1 (*M* = 1.99, *SD* = 0.49), *t* = 3.20, *p* < 0.05.

It seems that the most extreme ratings of attractiveness were given to target faces presented at position 1 or 5. Thus, it is plausible to regard positions 2, 3, and 4 as the central positions, and positions 1 and 5 as side positions. A 2 {[attractiveness (high vs. low)] × 2 [position (the central position vs. the side position)]} repeated-measure ANOVA was conducted on the mean ratings of attractiveness of the faces to examine the interaction effect. The results show that this effect was significant, *F*(1, 27) = 17.22, *p* < 0.001, *η* ^2^ = 0.39. The analysis of the main effect showed that attractive faces became less attractive when located at the center, *F*(1, 27) = 9.18, *p* < 0.01, and unattractive faces becoming more attractive when presented at the central position, *F*(1, 27) = 8.60, *p* < 0.01.

## Effect of Duration

We wanted to determine if the duration of presentation has an effect on facial attractiveness when the target face is presented in various positions (on the sides or in the center) within a lineup or by itself. We used independent sample *t*-tests to compare the data of Experiments 1 and 3 to this end.

Table 1 shows the effects of the duration of viewing on the perceived attractiveness of the most attractive and unattractive target faces. The attractiveness ratings of the unattractive target faces presented alone for 500 ms were higher than when presented for 1,000 ms, *t* = 2.12, *p* < 0.05. However, the ratings of the attractive faces presented at the center of the group for 500 ms are lower than those presented for 1,000 ms, *t* = −2.22, *p* < 0.05. The ratings of attractive faces presented alone for 500 ms were lower than when they were presented for 1,000 ms, *t* = −3.19, *p* < 0.01. There was no significant difference under the other conditions.

**Table 1:** Independent sample *t*-test (500 vs. 1,000).

	Test	Statistic	*df*	*p*	Cohen’s *d*
LowSide	Student	0.527	56.00	0.600	0.138
	Welch	0.527	55.52	0.601	0.138
LowMiddle	Student	0.363	56.00	0.718	0.095
	Welch	0.365	55.74	0.716	0.096
LowSingle	Student	2.115	56.00	0.039	0.556
	Welch	2.112	55.25	0.039	0.555
HighSide	Student	−1.826	56.00	0.073	−0.480
	Welch	−1.826	55.70	0.073	−0.480
HighMiddle	Student	−2.217	56.00	0.031	−0.583
	Welch	−2.209	54.26	0.031	−0.581
HighSingle	Student	−3.190	56.00	0.002^a^	−0.838
	Welch	−3.235	50.31	0.002	−0.844

a Levene’s test is significant (*p* < 0.05), suggesting a violation of the equal variance assumption.

For unattractive faces presented alone, a longer duration of viewing lowered attractiveness ratings. However, for attractive faces presented alone or at the center of a lineup, a longer duration of viewing improved the ratings.

These results indicate that people prefer to look at attractive faces for longer.

## Meta-Analysis Across Experiments

As recommended when multiple experiments include tests of the same effect (Maner, 2014), we used a single-paper meta-analysis (SPM; McShane and Böckenholt, 2017), which is a method to summarize multiple experiments by conducting a significance test of aggregated effects across them.

**Figure 6:**
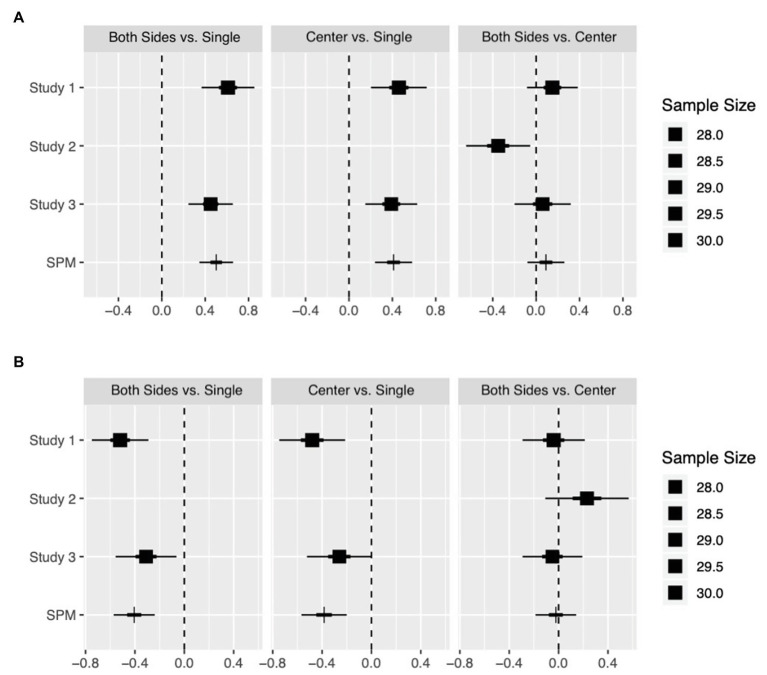
Effect estimates are given by the squares for single-study estimates and the vertical bars for single-paper meta-analysis estimates (SPM). The 50 and 95% intervals are represented by the thick and thin lines, respectively. The average sample size per condition in each study is given by the size of the squares. **(A)** Presents a comparison of highly attractive faces and **(B)** presents a comparison of highly unattractive faces under different conditions.

We tested three major contrasts in this meta-analysis. First, we compared the attractiveness of the target faces in different positions in a group (on the sides or at the center) with that by themselves. Second, we compared the attractiveness of the target faces when presented in different positions in a group (on the sides or at the center). We aggregated across all experiments to show the differences in attractiveness in different positions of presentation (in Figure 6). The SPM had much narrower uncertainty intervals than those of the single-study estimates by combining information across experiments.

The resulting estimates were more credible. The most attractive face in a group was perceived more attractive than when presented by itself regardless of its position in the group. Similarly, the most unattractive face in a group was perceived to be less attractive than in isolation regardless of its position in the group. These results provide reliable evidence of contrast effects.

However, the SPM estimates show that the differences in attractiveness between positions of presentation in a lineup were not significant, probably because of the different experimental procedures of the three experiments. The influential factors should be investigated further in future work.

## General Discussion

This study manipulated three variables (facial attractiveness, position of presentation, and duration of presentation) to investigate the differences in scores of different, attractive faces in different situations. The results showed that compared with independent presentation, the evaluation of the attractiveness of special faces in a group was more extreme, showing a contrast effect. The position of presentation had an effect in a group on the perceived attractiveness of the target face. In addition, the duration of viewing differently influenced the observers’ evaluations of attractive and unattractive target faces. The results indicate that the processing of the facial attractiveness of a group did not occur entirely according to ensemble coding.

The results of Experiment 1 show that when the duration of presentation was 500 ms, the subjects’ attractiveness ratings of highly attractive faces presented in a group were higher than when they were presented alone, but the ratings of unattractive faces presented in a group were lower. In Experiment 2, observers in middle group were asked to focus on the middle. The results show that the position of presentation had a significant impact on the judgment of facial attractiveness in a group. The subjects assigned higher scores to highly attractive faces in the middle of the group, but no significant difference was observed in the evaluation of unattractive faces in the middle. In Experiment 3, with an increase in the duration of viewing, position was found to affect the ratings of unattractive target faces. This confirms our explanation that people did not attend much too unattractive faces. When the duration of viewing was 500 ms, the effect of position on unattractive faces was not significant, but when the duration was 1,000 ms, the observers had more time to observe the unattractive target face in the group, and their ratings changed with the position of the face. The target face in the central position assimilated with the attractive surrounding faces. Therefore, a central attractive face was perceived to be less attractive, and a central unattractive face was perceived to be more attractive. A comparison of Experiments 1 and 3 suggests an effect of duration on the perceived attractiveness. People prefer seeing attractive faces for longer, but not unattractive faces.

The results of Experiments 1 and 3 showed that compared with when presented alone, the attractive faces when presented in groups were perceived to be even more attractive while unattractive faces presented in groups were perceived to be even less attractive. This supports the contrast effect. Researchers have observed the cheerleader effect or friend effect, whereby faces in a group are considered more attractive than when shown alone (Walker and Vul, 2014). This is partially incongruent with our findings for unattractive faces. In Experiments 1 and 3, compared with when presented alone, the perceived attractiveness of unattractive target faces in a group decreased, which suggests that unattractive faces in the presence of average attractive faces were considered more unattractive. When people observed these “special faces” in a group featuring significant differences in attractiveness among faces, they made more extreme evaluations, showing a contrast effect. This effect occurred when the duration of presentation was 500 ms. The results also showed that the subjects’ visual processing of group faces was not always ensemble coding-based processing, and in special cases, individual priority processing might have been carried out. Researchers who support individual priority processing claim that ensemble coding does not participate in the process of perception. Although the common characteristics of groups play a primary role in visual processing, the ensemble coding process cannot explain the principle of processing of the effect of group attractiveness (van Osch et al., 2015).

The attractiveness ratings of each face in a group were affected by the attractiveness of the surrounding faces. When three types of faces with different degrees of attractiveness were presented at the same time, the ratings of unattractive faces were lower and those ratings of highly attractive faces were higher, showing a contrast effect (Furl, 2016). Furl (2016) claimed that when people judge the target stimulus, they always compare it with the environment, which serves as reference for people to judge the stimulus. Our results supported Furl’s views. Faces that were highly attractive or unattractive had a contrast effect with those around them. But we also noted greater assimilation when the target face was at the center. It is easier to compare a face with surrounding faces when it is in the center; thus, context has a significant influence on the central face.

Ying et al. (2019) suggested that the cheerleader effect consists of a contrastive effect and a social positive effect. The social positive effect refers to a target face being more attractive when in a group regardless of the attractiveness of the group. In our experiments, the results for attractive faces replicated these findings using a different research paradigm. However, the lower attractiveness ratings of unattractive faces in groups do not support the social positive effect. What causes the social positive effect remains unclear. There is debate on the mechanisms underlying the cheerleader effect, which is observed even though it is incompatible with hierarchical encoding (Carragher et al., 2019). This offers limited evidence for the role of hierarchical encoding in the cheerleader effect (Luo and Zhou, 2018). It is important to re-examine the underlying causes of the cheerleader effect in future research.

The results of Experiment 2 support the middle effect. The middle may have an impact on the attractiveness score of the target face. Another study found that the subjects only had higher social evaluation on the highly attractive faces, such as “beautiful is good,” but not on the highly unattractive faces (Zebrowitz and Franklin, 2014). Therefore, the subjects only had higher evaluation on the highly attractive faces in the middle, while there was no significant increase on the highly unattractive faces in the middle, which was showed in Experiment 2.

Interestingly, the effect of position on facial attractiveness in Experiment 3 differed from that in Experiment 2. In Experiment 2, the attractiveness of an attractive face in the middle was higher than in other positions, whereas in Experiment 3, the attractiveness of an attractive face in the center was lower than in other positions, but unattractive faces were perceived to be more attractive when in the center. We assume that the differences between the experimental procedures led to this difference.

In Experiment 2, participants in the Middle group were probably more prepared to rate the face in the center. They likely fixed their gaze at the center. The long-time fixation on the central face and implicit assumption of its superior status increased the perceived attractiveness. But in Experiment 3, participants could not have their eyes on the middle and need to observe all five positions to find the target. Compared with Experiment 2, participants in Experiment 3 were aware of more contextual information, which caused the greater influence on rating the target face.

We suggest that when the target face appeared in the center, the association between the central face and the group was stronger, leading to an assimilation effect. The evaluation of the central face was assimilated in the attractiveness of the surrounding faces, and the face in the middle was rated less attractive (Geiselman et al., 1984). This is in line with observers’ bias toward the average attractiveness of the group by implicitly extracting information on it, when the target face is surrounded by a group of faces with heterogeneous degrees of attractiveness (Luo and Zhou, 2018). Rodway et al. (2013) found both greater assimilation and greater contrast, with attractive faces perceived less attractive regardless of the attractiveness of the surrounding faces, and unattractive faces perceived more attractive only when flanked by other unattractive faces. Interestingly, in Experiment 3, the assimilation effect also occurred, whereby the attractiveness ratings of an unattractive face increased when it was flanked by average attractive faces.

In contrast to this study, Carragher et al. (2018) found that the face is perceived to be more attractive in a group regardless of its location in it. They used a direct-rating paradigm. The errors might involve in the measurement by this subjective way. Their experimental stimuli and procedure were different from ours, which might have led to the inconsistency. In our experiments, the screen showed five faces in a group. However, in their study, only three faces were shown on the screen. It is easier to perceive the attractiveness of all three faces than to perceive the attractiveness of all five faces, which results in the no effect of position on attractiveness in their research. The faces in our experiments were processed to a consistent gray level, leaving only facial features with neutral emotions, whereas Carragher et al. (2018) used pictures retaining the background and hair along with positive expressions. It is likely that people pay more attention on the uncontrolled background and hair rather than on the attractiveness of faces. And observers may look at the person with bright hair and glasses firstly though she may not be the most attractive in the group. The confounding factors could affect observers’ evaluations of facial attractiveness. Importantly, they did not differentiate in terms of attractiveness between the target face and other faces in a group, whereas we did. In our study, the target faces were the most and least attractive faces while the “distractors” were faces with average attractiveness as rated by the participants. In research of Carragher et al. (2018), the difference in facial attractiveness between the target and the distractors was not significant, and faces with the same expressions could easily be regarded as a group of close friends, thus leading to the cheerleader effect. They found strong evidence in support of the cheerleader effect regardless of the spatial configuration of the group. And we found the position effect on facial attractiveness. It is interesting to explore whether the position effect is more significant when increasing the number of members in groups and making each member appear the same (the same hair and skin color).

We found that for special faces presented in a group, compared with when presented alone, a contrast effect was observed in the assessment. That is, subjects assigned higher scores to highly attractive target faces and lower ones to highly unattractive ones. When the target face was shown at the center of a group, the assimilation effect occurred. The results further showed that ensemble coding-based processing was not the only processing mechanism in assessing the facial attractiveness of a group, and the subjects might have carried out “individual priority processing” under specific conditions.

We think that there is a contrast effect for faces with the extreme attractiveness in a group when the observers had some contextual information. When they had more contextual information, the assimilation effect occurred. Future research should be conducted to determine the precise situation that triggers the contrast effect or the assimilation effect.

The ensemble perception of a group may have important evolutionary significance as people can compare facial attractiveness on a group basis (Phillips et al., 2014). In addition, our judgment of a member is influenced by how the overall attractiveness of a social group impresses us. The attractiveness of a face depends on context. Indeed, people may even socially assess a person differently according to the people surrounding him/her and location.

This study has some shortcomings. First, the subjects selected in the study were college students and employees at a university. Owing to their large age range, faces might have had different aesthetic appeal for them. Second, the face images were selected from the Internet, and only the facial features were retained when processing them. The processed images were different from actual faces (no hair and face contours), which might have had an impact on the aesthetic judgments of the subjects.

Future research in the area should focus on the following: first, age should be considered an independent variable for a vertical comparison to explore the differences in the evaluations of the attractiveness of the target faces among subjects of different ages who may prefer faces with different characteristics. Second, the influence of special faces in a group on the perceived attractiveness of other members should be explored. We expect applications of neuroimaging, such as eye-tracking, to help identify distinct stages of cognition responsible for driving ensemble statistics.

## Conclusions

According to the results of this study, the following conclusions can be preliminarily drawn: compared with independent presentation, the subjects exhibited a contrast effect in assessing the attractiveness of special faces in a group, which shows that people’s mode of the visual processing of a group was not always ensemble coding-based processing. The position of presentation has a significant impact on the judgment of attractiveness of special faces in a group. Contrast and assimilation occur at the same time depending on their association with the context.

## Data Availability Statement

The datasets generated for this study are available on request to the corresponding author.

## Ethics Statement

The studies involving human participants were reviewed and approved by School of Psychology, South China Normal University. The patients/participants provided their written informed consent to participate in this study.

## Author Contributions

ZT and TZ designed the study and collected a part of data. YL collected the rest of data. ZT, YL, and TZ performed the data analyses. YL and XH approved the manuscript for submission. XH supervised the entire project. All authors contributed to the article and approved the submitted version.

### Conflict of Interest

The authors declare that the research was conducted in the absence of any commercial or financial relationships that could be construed as a potential conflict of interest.
