# Supplementary figures and images for: Contrast Effect of Facial Attractiveness in Groups
Source: Front Psychol. 2020 Sep 15;11:2258. doi: 10.3389/fpsyg.2020.02258 (PMC7523431; doi:10.3389/fpsyg.2020.02258)

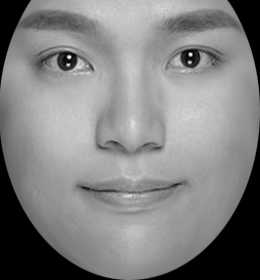

Supplement: Supplementary file 1 [file presentation_1.zip › stimuli/male/attractive/M113.bmp]

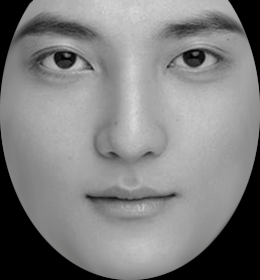

Supplement: Supplementary file 1 [file presentation_1.zip › stimuli/male/attractive/M92.bmp]

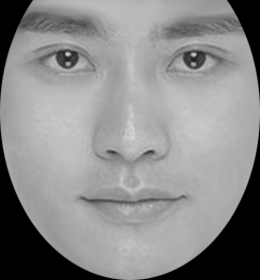

Supplement: Supplementary file 1 [file presentation_1.zip › stimuli/male/attractive/M106.bmp]

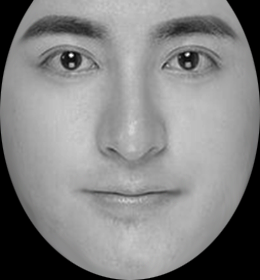

Supplement: Supplementary file 1 [file presentation_1.zip › stimuli/male/attractive/M112.bmp]

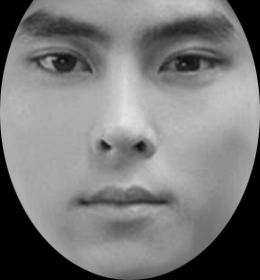

Supplement: Supplementary file 1 [file presentation_1.zip › stimuli/male/attractive/M104.bmp]

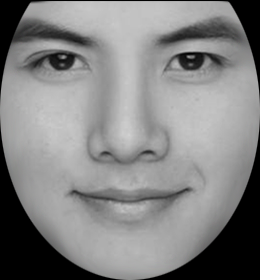

Supplement: Supplementary file 1 [file presentation_1.zip › stimuli/male/attractive/M91.bmp]

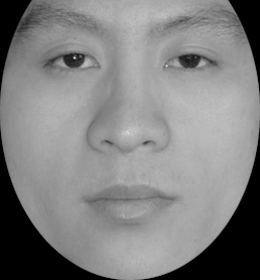

Supplement: Supplementary file 1 [file presentation_1.zip › stimuli/male/attractive/M47.bmp]

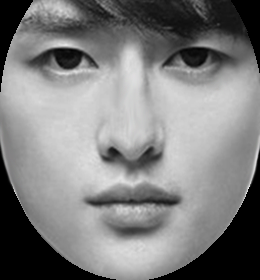

Supplement: Supplementary file 1 [file presentation_1.zip › stimuli/male/attractive/M139.bmp]

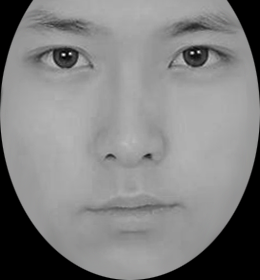

Supplement: Supplementary file 1 [file presentation_1.zip › stimuli/male/attractive/M101.bmp]

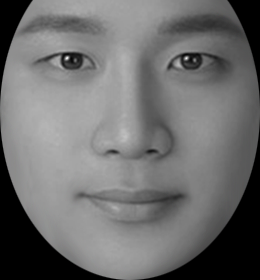

Supplement: Supplementary file 1 [file presentation_1.zip › stimuli/male/attractive/M100.bmp]

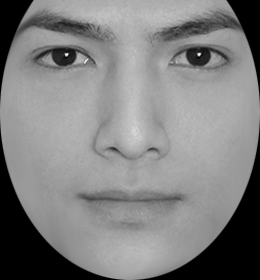

Supplement: Supplementary file 1 [file presentation_1.zip › stimuli/male/attractive/M97.bmp]

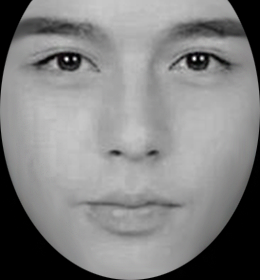

Supplement: Supplementary file 1 [file presentation_1.zip › stimuli/male/attractive/M96.bmp]

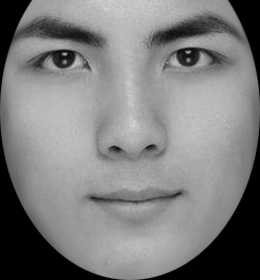

Supplement: Supplementary file 1 [file presentation_1.zip › stimuli/male/attractive/M103.bmp]

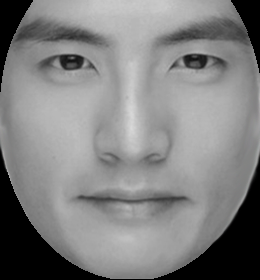

Supplement: Supplementary file 1 [file presentation_1.zip › stimuli/male/attractive/M148.bmp]

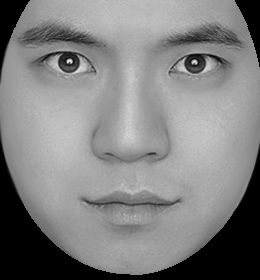

Supplement: Supplementary file 1 [file presentation_1.zip › stimuli/male/attractive/M160.bmp]

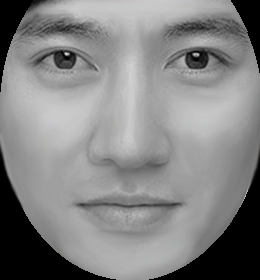

Supplement: Supplementary file 1 [file presentation_1.zip › stimuli/male/attractive/M150.bmp]

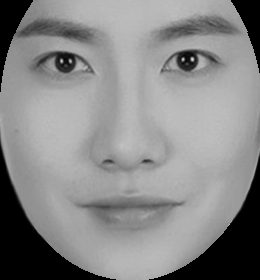

Supplement: Supplementary file 1 [file presentation_1.zip › stimuli/male/attractive/M153.bmp]

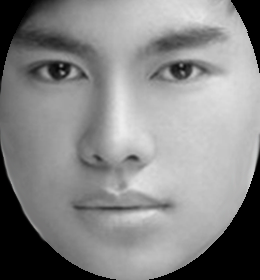

Supplement: Supplementary file 1 [file presentation_1.zip › stimuli/male/attractive/M143.bmp]

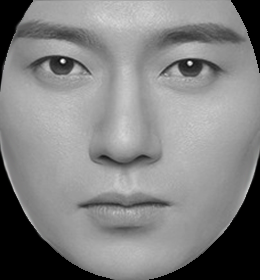

Supplement: Supplementary file 1 [file presentation_1.zip › stimuli/male/attractive/M156.bmp]

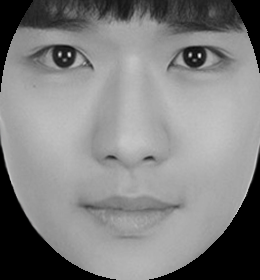

Supplement: Supplementary file 1 [file presentation_1.zip › stimuli/male/attractive/M154.bmp]

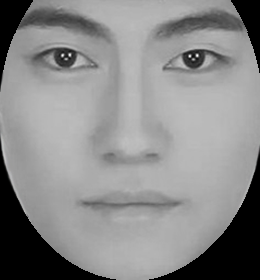

Supplement: Supplementary file 1 [file presentation_1.zip › stimuli/male/attractive/M155.bmp]

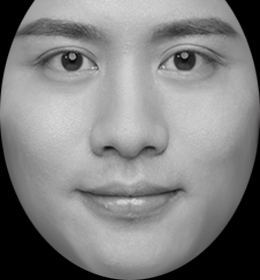

Supplement: Supplementary file 1 [file presentation_1.zip › stimuli/male/attractive/M119.bmp]

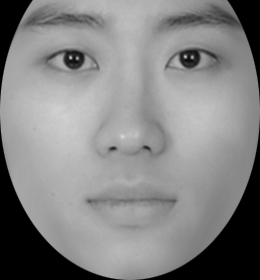

Supplement: Supplementary file 1 [file presentation_1.zip › stimuli/male/attractive/M125.bmp]

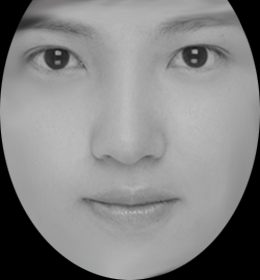

Supplement: Supplementary file 1 [file presentation_1.zip › stimuli/male/attractive/M118.bmp]

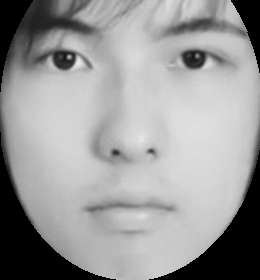

Supplement: Supplementary file 1 [file presentation_1.zip › stimuli/male/attractive/M136.bmp]

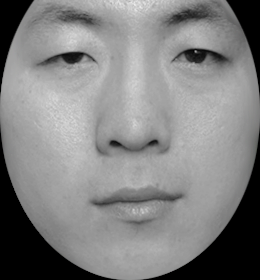

Supplement: Supplementary file 1 [file presentation_1.zip › stimuli/male/unattractive/M87.bmp]

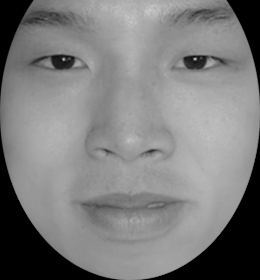

Supplement: Supplementary file 1 [file presentation_1.zip › stimuli/male/unattractive/M78.bmp]

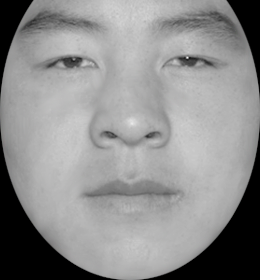

Supplement: Supplementary file 1 [file presentation_1.zip › stimuli/male/unattractive/M50.bmp]

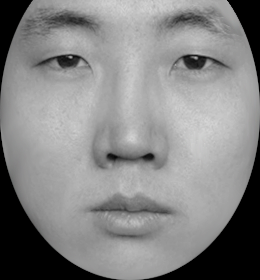

Supplement: Supplementary file 1 [file presentation_1.zip › stimuli/male/unattractive/M56.bmp]

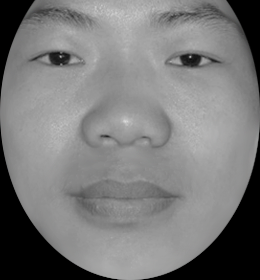

Supplement: Supplementary file 1 [file presentation_1.zip › stimuli/male/unattractive/M68.bmp]

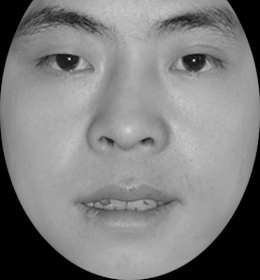

Supplement: Supplementary file 1 [file presentation_1.zip › stimuli/male/unattractive/M83.bmp]

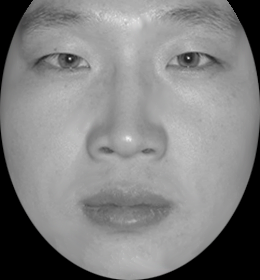

Supplement: Supplementary file 1 [file presentation_1.zip › stimuli/male/unattractive/M41.bmp]

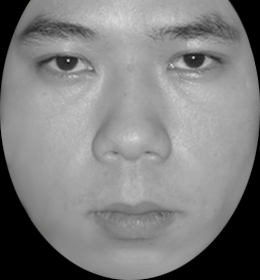

Supplement: Supplementary file 1 [file presentation_1.zip › stimuli/male/unattractive/M32.bmp]

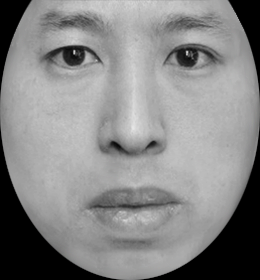

Supplement: Supplementary file 1 [file presentation_1.zip › stimuli/male/unattractive/M27.bmp]

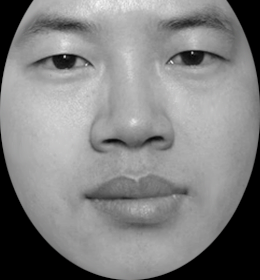

Supplement: Supplementary file 1 [file presentation_1.zip › stimuli/male/unattractive/M24.bmp]

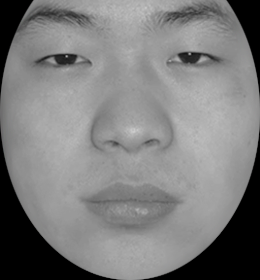

Supplement: Supplementary file 1 [file presentation_1.zip › stimuli/male/unattractive/M37.bmp]

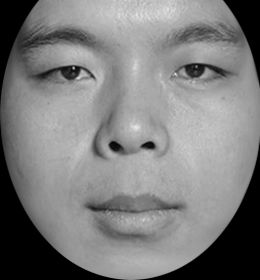

Supplement: Supplementary file 1 [file presentation_1.zip › stimuli/male/unattractive/M8.bmp]

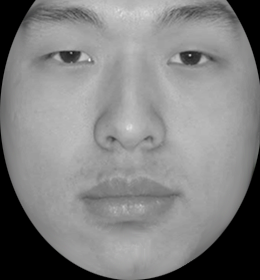

Supplement: Supplementary file 1 [file presentation_1.zip › stimuli/male/unattractive/M36.bmp]

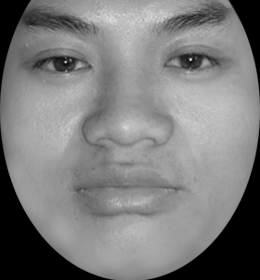

Supplement: Supplementary file 1 [file presentation_1.zip › stimuli/male/unattractive/M22.bmp]

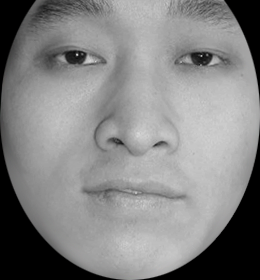

Supplement: Supplementary file 1 [file presentation_1.zip › stimuli/male/unattractive/M4.bmp]

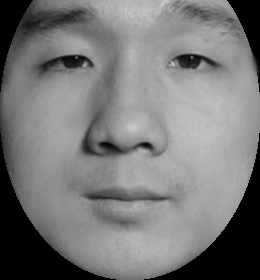

Supplement: Supplementary file 1 [file presentation_1.zip › stimuli/male/unattractive/M10.bmp]

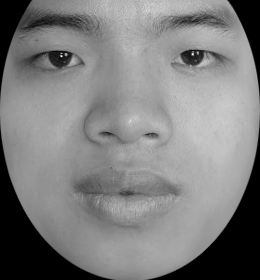

Supplement: Supplementary file 1 [file presentation_1.zip › stimuli/male/unattractive/M11.bmp]

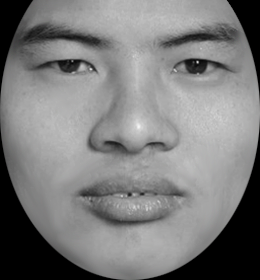

Supplement: Supplementary file 1 [file presentation_1.zip › stimuli/male/unattractive/M2.bmp]

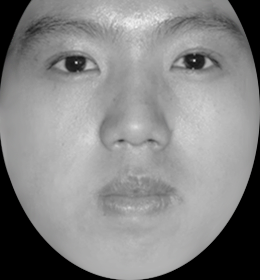

Supplement: Supplementary file 1 [file presentation_1.zip › stimuli/male/unattractive/M70.bmp]

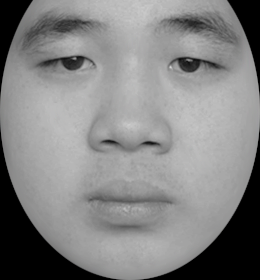

Supplement: Supplementary file 1 [file presentation_1.zip › stimuli/male/unattractive/M58.bmp]

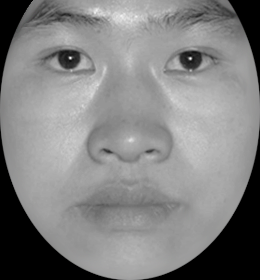

Supplement: Supplementary file 1 [file presentation_1.zip › stimuli/male/unattractive/M71.bmp]

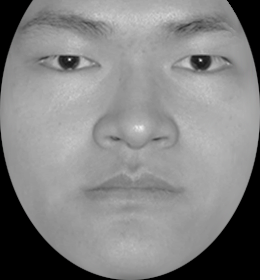

Supplement: Supplementary file 1 [file presentation_1.zip › stimuli/male/unattractive/M72.bmp]

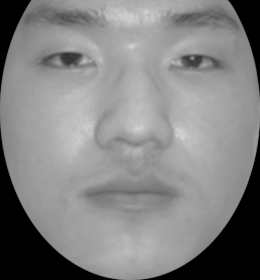

Supplement: Supplementary file 1 [file presentation_1.zip › stimuli/male/unattractive/M89.bmp]

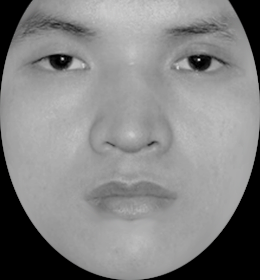

Supplement: Supplementary file 1 [file presentation_1.zip › stimuli/male/unattractive/M77.bmp]

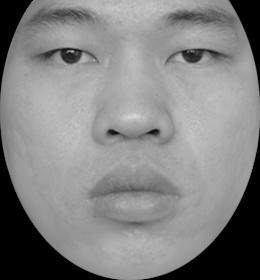

Supplement: Supplementary file 1 [file presentation_1.zip › stimuli/male/unattractive/M60.bmp]

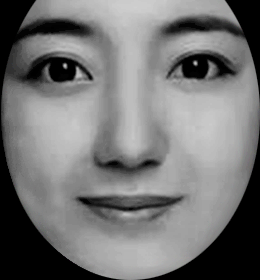

Supplement: Supplementary file 1 [file presentation_1.zip › stimuli/female/attractive/F126.bmp]

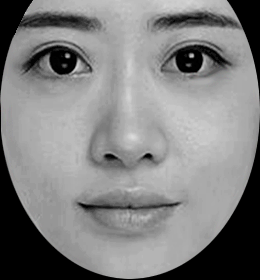

Supplement: Supplementary file 1 [file presentation_1.zip › stimuli/female/attractive/F127.bmp]

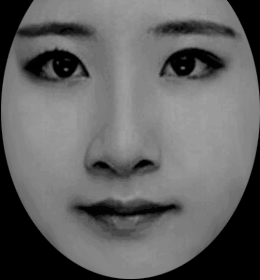

Supplement: Supplementary file 1 [file presentation_1.zip › stimuli/female/attractive/F125.bmp]

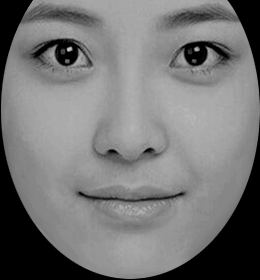

Supplement: Supplementary file 1 [file presentation_1.zip › stimuli/female/attractive/F124.bmp]

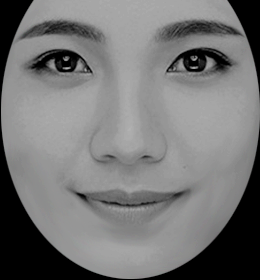

Supplement: Supplementary file 1 [file presentation_1.zip › stimuli/female/attractive/F109.bmp]

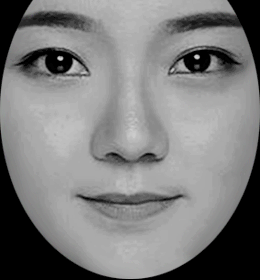

Supplement: Supplementary file 1 [file presentation_1.zip › stimuli/female/attractive/F135.bmp]

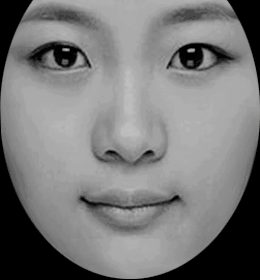

Supplement: Supplementary file 1 [file presentation_1.zip › stimuli/female/attractive/F136.bmp]

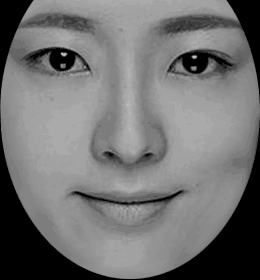

Supplement: Supplementary file 1 [file presentation_1.zip › stimuli/female/attractive/F151.bmp]

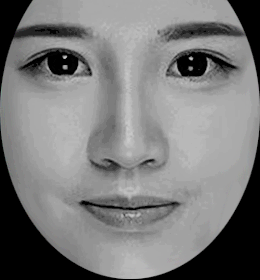

Supplement: Supplementary file 1 [file presentation_1.zip › stimuli/female/attractive/F145.bmp]

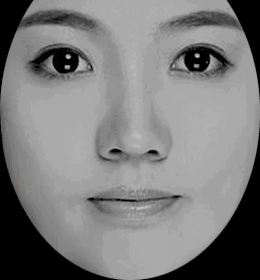

Supplement: Supplementary file 1 [file presentation_1.zip › stimuli/female/attractive/F146.bmp]

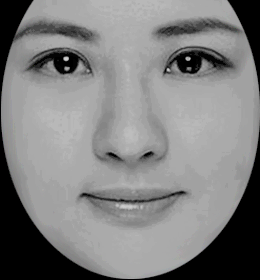

Supplement: Supplementary file 1 [file presentation_1.zip › stimuli/female/attractive/F147.bmp]

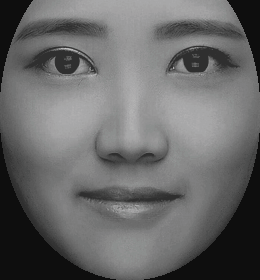

Supplement: Supplementary file 1 [file presentation_1.zip › stimuli/female/attractive/F154.bmp]

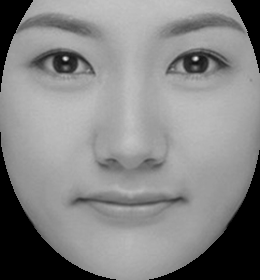

Supplement: Supplementary file 1 [file presentation_1.zip › stimuli/female/attractive/F165.bmp]

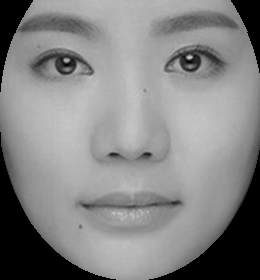

Supplement: Supplementary file 1 [file presentation_1.zip › stimuli/female/attractive/F159.bmp]

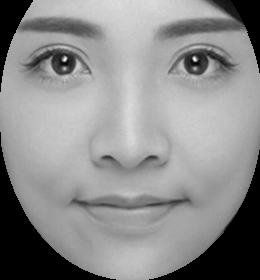

Supplement: Supplementary file 1 [file presentation_1.zip › stimuli/female/attractive/F172.bmp]

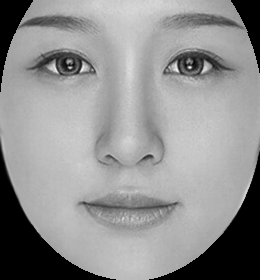

Supplement: Supplementary file 1 [file presentation_1.zip › stimuli/female/attractive/F166.bmp]

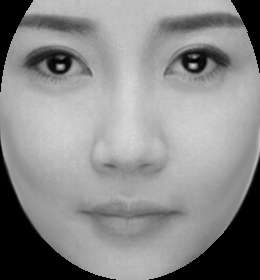

Supplement: Supplementary file 1 [file presentation_1.zip › stimuli/female/attractive/F160.bmp]

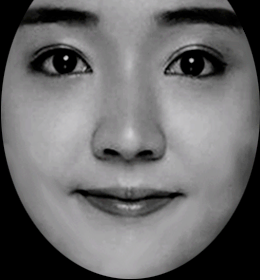

Supplement: Supplementary file 1 [file presentation_1.zip › stimuli/female/attractive/F106.bmp]

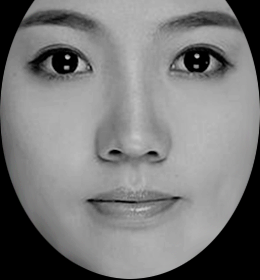

Supplement: Supplementary file 1 [file presentation_1.zip › stimuli/female/attractive/F111.bmp]

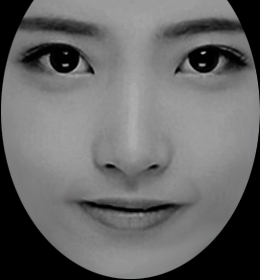

Supplement: Supplementary file 1 [file presentation_1.zip › stimuli/female/attractive/F101.bmp]

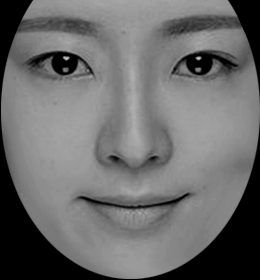

Supplement: Supplementary file 1 [file presentation_1.zip › stimuli/female/attractive/F115.bmp]

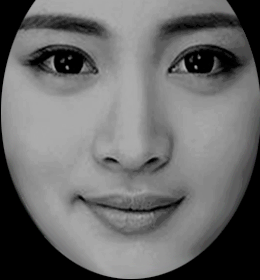

Supplement: Supplementary file 1 [file presentation_1.zip › stimuli/female/attractive/F129.bmp]

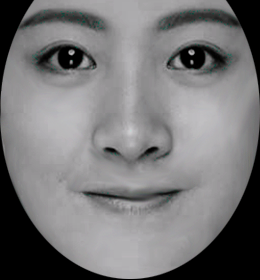

Supplement: Supplementary file 1 [file presentation_1.zip › stimuli/female/attractive/F102.bmp]

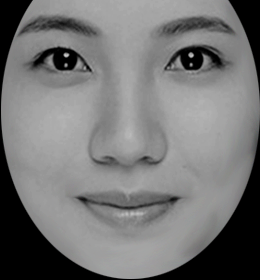

Supplement: Supplementary file 1 [file presentation_1.zip › stimuli/female/attractive/F103.bmp]

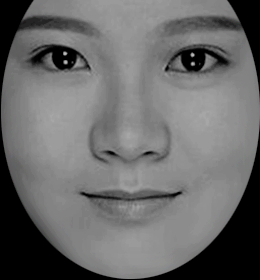

Supplement: Supplementary file 1 [file presentation_1.zip › stimuli/female/attractive/F117.bmp]

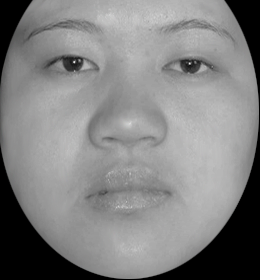

Supplement: Supplementary file 1 [file presentation_1.zip › stimuli/female/unattractive/F39.bmp]

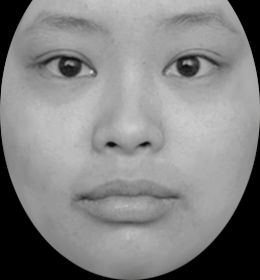

Supplement: Supplementary file 1 [file presentation_1.zip › stimuli/female/unattractive/F10.bmp]

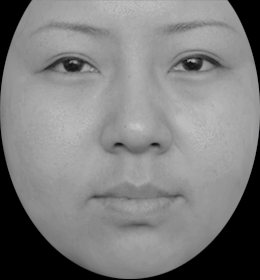

Supplement: Supplementary file 1 [file presentation_1.zip › stimuli/female/unattractive/F14.bmp]

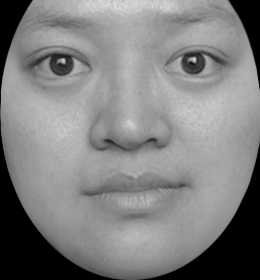

Supplement: Supplementary file 1 [file presentation_1.zip › stimuli/female/unattractive/F71.bmp]

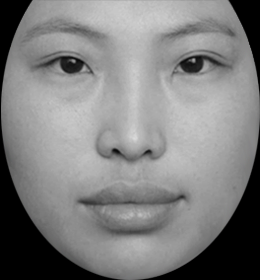

Supplement: Supplementary file 1 [file presentation_1.zip › stimuli/female/unattractive/F64.bmp]

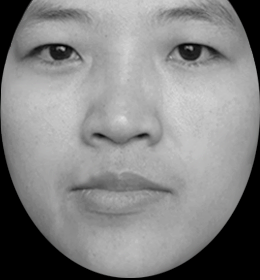

Supplement: Supplementary file 1 [file presentation_1.zip › stimuli/female/unattractive/F72.bmp]

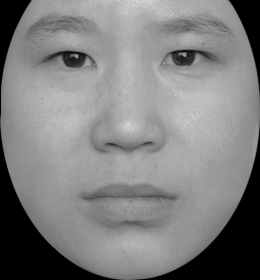

Supplement: Supplementary file 1 [file presentation_1.zip › stimuli/female/unattractive/F62.bmp]

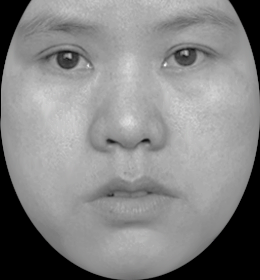

Supplement: Supplementary file 1 [file presentation_1.zip › stimuli/female/unattractive/F61.bmp]

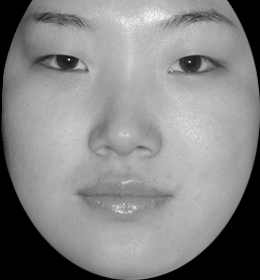

Supplement: Supplementary file 1 [file presentation_1.zip › stimuli/female/unattractive/F78.bmp]

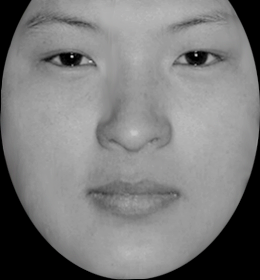

Supplement: Supplementary file 1 [file presentation_1.zip › stimuli/female/unattractive/F44.bmp]

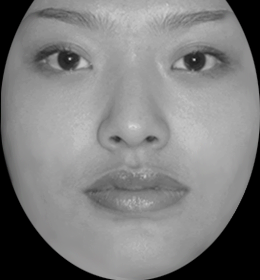

Supplement: Supplementary file 1 [file presentation_1.zip › stimuli/female/unattractive/F90.bmp]

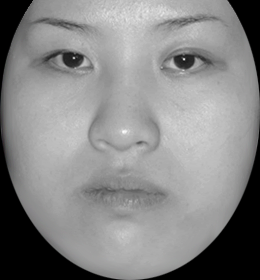

Supplement: Supplementary file 1 [file presentation_1.zip › stimuli/female/unattractive/F84.bmp]

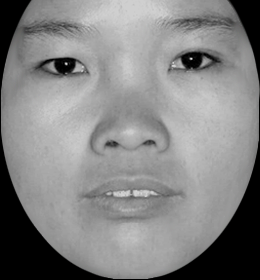

Supplement: Supplementary file 1 [file presentation_1.zip › stimuli/female/unattractive/F52.bmp]

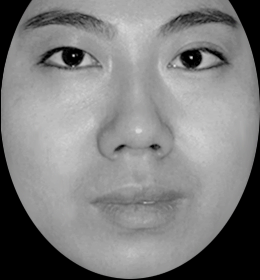

Supplement: Supplementary file 1 [file presentation_1.zip › stimuli/female/unattractive/F57.bmp]

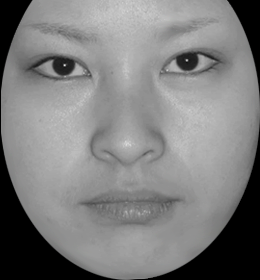

Supplement: Supplementary file 1 [file presentation_1.zip › stimuli/female/unattractive/F82.bmp]

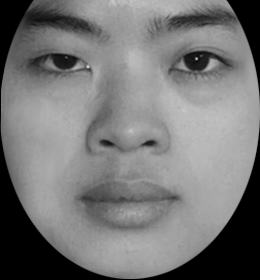

Supplement: Supplementary file 1 [file presentation_1.zip › stimuli/female/unattractive/F41.bmp]

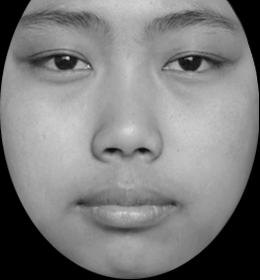

Supplement: Supplementary file 1 [file presentation_1.zip › stimuli/female/unattractive/F33.bmp]

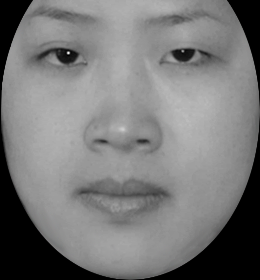

Supplement: Supplementary file 1 [file presentation_1.zip › stimuli/female/unattractive/F32.bmp]

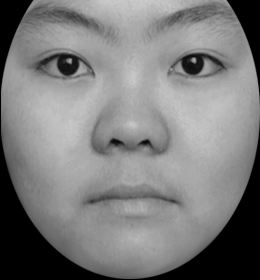

Supplement: Supplementary file 1 [file presentation_1.zip › stimuli/female/unattractive/F3.bmp]

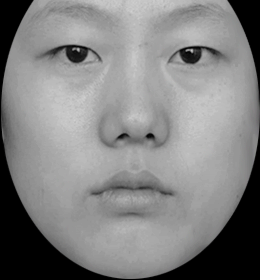

Supplement: Supplementary file 1 [file presentation_1.zip › stimuli/female/unattractive/F18.bmp]

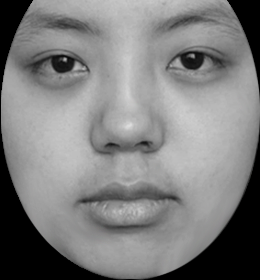

Supplement: Supplementary file 1 [file presentation_1.zip › stimuli/female/unattractive/F31.bmp]

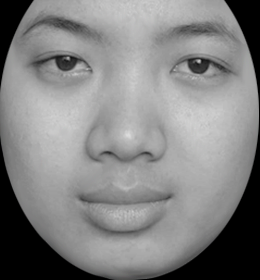

Supplement: Supplementary file 1 [file presentation_1.zip › stimuli/female/unattractive/F35.bmp]

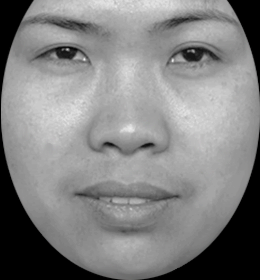

Supplement: Supplementary file 1 [file presentation_1.zip › stimuli/female/unattractive/F21.bmp]

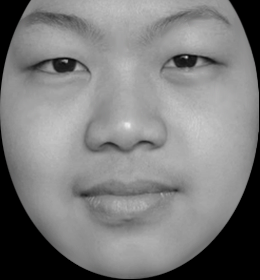

Supplement: Supplementary file 1 [file presentation_1.zip › stimuli/female/unattractive/F34.bmp]

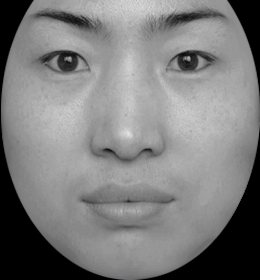

Supplement: Supplementary file 1 [file presentation_1.zip › stimuli/female/unattractive/F22.bmp]
